# Panmixia and dispersal from the Mediterranean Basin to Macaronesian Islands of a macrolichen species

**DOI:** 10.1038/srep40879

**Published:** 2017-01-19

**Authors:** David Alors, Francesco Dal Grande, Paloma Cubas, Ana Crespo, Imke Schmitt, M. Carmen Molina, Pradeep K. Divakar

**Affiliations:** 1 Departamento de Biología Vegetal II, Facultad de Farmacia, Universidad Complutense de Madrid, 28040 Madrid, Spain; 2 Senckenberg Biodiversity and Climate Research Centre (BiK-F), Senckenberganlage 25, 60325 Frankfurt am Main, Germany; 3 Institute of Ecology, Evolution and Diversity, Goethe Universität, Max-von-Laue-Str. 13, D-60438 Frankfurt, Germany; 4 Departamento de Biología y Geología, Física y Química Inorgánica (Área de Biodiversidad y Conservación), ESCET, Universidad Rey Juan Carlos, Móstoles, 28933 Madrid, Spain

## Abstract

The Mediterranean region, comprising the Mediterranean Basin and the Macaronesian Islands, represents a center of diversification for many organisms. The genetic structure and connectivity of mainland and island microbial populations has been poorly explored, in particular in the case of symbiotic fungi. Here we investigated genetic diversity and spatial structure of the obligate outcrossing lichen-forming fungus *Parmelina carporrhizans* in the Mediterranean region. Using eight microsatellite and mating-type markers we showed that fungal populations are highly diverse but lack spatial structure. This is likely due to high connectivity and long distance dispersal of fungal spores. Consistent with low levels of linkage disequilibrium and lack of clonality, we detected both mating-type idiomorphs in all populations. Furthermore we showed that the Macaronesian Islands are the result of colonization from the Mediterranean Basin. The unidirectional gene flow, though, seemed not to be sufficient to counterbalance the effects of drift, resulting in comparatively allelic poor peripheral populations. Our study is the first to shed light on the high connectivity and lack of population structure in natural populations of a strictly sexual lichen fungus. Our data further support the view of the Macaronesian Islands as the end of the colonization road for this symbiotic ascomycete.

Cryptic biodiversity is an essential component of biodiversity that will be considerably affected by global climate change in the next decades^1^. In recent years molecular genetics has shown that cryptic species are more common across organismal groups than previously thought^2^. While there is an increased awareness of the existence and importance of cryptic species, there is a general lack of studies on the amount and distribution of genetic diversity within and among populations of these lineages^3^. The future of a species, in terms of evolutionary potential, depends on its genetic diversity. This can be affected by the spatial distribution of the species as it influences the amount of gene flow among populations^4^. Understanding how genetic structure is spatially distributed, within and between populations, is important because it reflects the biology and history of species and determines their evolutionary potential^5^,^6^.

Lichen-forming fungi are a successful group of nutritionally specialized fungi that form obligate symbioses with photosynthetic partners. Molecular studies of lichen-forming fungi have repeatedly indicated that numerous distinct lineages, i.e. cryptic species, can be hidden under a single taxon. This shows that species diversity of lichen-forming fungi might be vastly underestimated, mainly because of the plasticity of morphological and chemical characters and the frequency of homoplasy and convergence of characters^7^,^8^,^9^. In the past decades, the use of DNA-based molecular phylogenies for unraveling the hidden diversity in lichen-forming fungi has become increasingly common. However, investigations of these cryptic lineages at the population level are still far less common^10^.

Lichen-forming fungi are interesting subjects for population genetics studies. One of the reasons for this is the vast range of reproductive strategies, i.e., from purely asexual to exclusively sexual, that could differentially shape their population structures^11^. Lichens may also have peculiar distributions, i.e. across contrasting environmental conditions, that could result in locally adapted populations. Further, sexual species can either be homothallic (self-fertile), i.e. carrying both required compatible mating types within the same individual, or heterothallic (outcrossing), i.e. requiring the fusion of two individuals with different mating types^12^. The expectation is that among sexual species, heterothallic species will harbor more genetic variability than self-fertile ones as each sexual reproductive event may generate new genotypes. Furthermore, exclusively sexual species should, in principle, exhibit less genetic structure than purely asexual ones because sexual spores are assumed to disperse over larger distances than asexual propagules, thus homogenizing genetic structures^11^,^13^. Overall, the effect will eventually depend on the relative frequency of sexuality over clonality, thus indirectly on the relative abundance of compatible mating types in populations for outcrossing species, and on the spatial dispersal range of the reproductive propagules^13^,^14^,^15^.

For investigating intra-population variability and inferring the role of reproductive strategies in shaping population dynamics, highly variable molecular markers such as microsatellites (SSRs) are one of the most appropriate tools, especially for highly clonal organisms such as lichens^16^. In particular, SSRs are suitable markers for studying hierarchically structured genetic diversity as their high level of polymorphism ensures a high yield of genetic variability^11^. However, only a few studies are available which have analyzed the population genetic structure of lichen-forming fungi with these markers. In the few species for which they have been utilized they have revealed high levels of intra-population polymorphism where traditional “variable” nuclear ribosomal markers (e.g., ITS and other ribosomal loci) had revealed little^17^,^18^. In the case of the exclusively sexual species *Buellia frigida*, a crustose lichen of continental Antarctica, an SSR-based study revealed high intra-population diversity, local population differentiation and limited dispersal, probably resulting from harsh habitat conditions and scarce spatial and temporal habitat availability^19^. Conversely, in the case of the predominantly asexual lichen-forming species *Lobaria pulmonaria*, SSR-based studies have shown highly clonal and structured populations, resulting from low level of recombination and limited dispersal capacity of vegetative propagules^20^,^21^. More pronounced population structure seems to be a common trend of vegetatively reproducing lichens as shown in several studies on other systems (e.g., *Parmotrema tinctorum*^22^, *Parmelina tiliacea*^23^). *Lobaria pulmonaria* is also the only lichen species for which population-level data on the distribution of mating-type idiomorphs is available so far^13^. This study showed a link between significantly unbalanced MAT-gene distribution and high levels of clonality in populations, i.e. lower intra-population genetic diversity. However, given the paucity of SSR-based studies on lichen-forming fungi with different reproductive strategies and the lack of studies on the distribution of mating types in natural populations, it is not possible to draw general conclusions for the effects of reproductive modes on lichen population structure.

In this study we analyzed intra-species genetic diversity, population structure, and mating-type distribution in natural populations of the obligate outcrossing lichen-forming fungal species *Parmelina carporrhizans*^24^*. Parmelina carporrhizans* is a foliose, epiphytic macrolichen with the center of distribution in the Mediterranean region. The Mediterranean region, comprising the Mediterranean Basin and the Macaronesian Islands, is an important centre of diversification for many plant and animal species^25^,^26^,^27^. However, in the case of lichen-forming fungi, the population structure and connectivity of mainland and island populations in this region have been largely unexplored. We thus aimed at exploring the predictions of two key hypotheses: i) the sexual reproductive mode of the species leads to high intra-population genetic diversity, high mainland-island connectivity and low population structure, and ii) high frequency of sexual reproduction is reflected in a balanced representation of mating-type idiomorphs in the populations.

To test these hypotheses, we used highly variable, species-specific microsatellite and mating-type specific markers. First, we analyzed the species population genetic diversity and evaluated how it is hierarchically and spatially partitioned. We assessed the extent to which alleles are geographically restricted or shared across regions. Second, we calculated the relative abundance of the two mating-type idiomorphs in the populations. Self-incompatible species require the presence of both mating types in the population for sexual reproduction. Third, we estimated migration rates among mainland and island groups. Combining genetic diversity and gene flow estimates we inferred whether the distribution of *P. carporrhizans* is a result of migration from the Macaronesian Islands to the continent or from the continent to the Macaronesian Islands.

## Results

### Mating-type idiomorph distribution

We successfully amplified the MAT-genes fragments in 93% of the samples. In each sample we found a single band corresponding to one idiomorph (Fig. S1, Supporting Information). These results are consistent with a heterothallic organization of MAT genes in *P. carporrhizans*.

The overall difference in frequency of the mating-type idiomorphs was not significantly different, with MAT1-1 found in 55% of the samples, and MAT1-2 in 45% of the samples. Only two populations (Morocco and Herbés) had a significantly skewed higher frequency of MAT1-1 idiomorphs (Table 1).

### Genotypic diversity

All eight microsatellite loci were highly polymorphic. The analysis of 220 samples with eight microsatellite markers resulted in a total of 220 microsatellite alleles, ranging from 16 alleles (Pcar1) to 46 alleles (Pcar7) (Table S1). No identical multilocus genotypes (MLGs) were found between individuals. The mean frequency of missing data per locus was 3.0% with a range of 0.4% (Pcar2) to 10% (Pcar8) (Table S1).

Overall we found a high unbiased haploid diversity (mean uh = 0.846), ranging from 0.744 (Tenerife) to 0.898 (Sicily). Populations had high population allelic richness (mean = 5.244), ranging from 3.973 (Cádiz) to 6.047 (Gredos). Private allelic richness was moderately low in all populations, ranging from 0.447 (Tenerife) to 1.072 (Gredos). At the regional level, the Macaronesian Islands had lower allelic (11.486) and private (3.095) allelic richness than the mainland populations (19.067 and 10.676, respectively) (Table 1).

We detected low levels of linkage disequilibrium (LD) across populations (mean = 0.069). Low but significant LD values were found in four populations, namely Gran Canaria 1 (rBarD = 0.045, *P* = 0.014), Cádiz (rBarD = 0.288, *P* = 0.001), Famalicão (rBarD = 0.043, *P* = 0.001) and Herbés (rBarD = 0.097, *P* = 0.002) (Table 1). The AMOVA analysis indicated that most of the molecular variation in *P. carporrhizans* populations occurs within populations (92.1%), with lesser amounts among populations within regions (2.3%) and among regions (5.6%) (Table 2). Permutation tests (based on 20,000 permutations) suggest that differences among populations (F_ST_ = 0.079) and regions (F_CT_ = 0.056) are not significant (*P* = 0.11 and 0.08, respectively).

As expected, pairwise F_ST_ values between populations were low, ranging from 0 to 0.023 (Table 3) and only two pairwise comparisons (Cádiz vs Famalicão and Gredos, both F_ST_ = 0.016) were statistically significant.

### Population genetic structure

**Figure 1:**
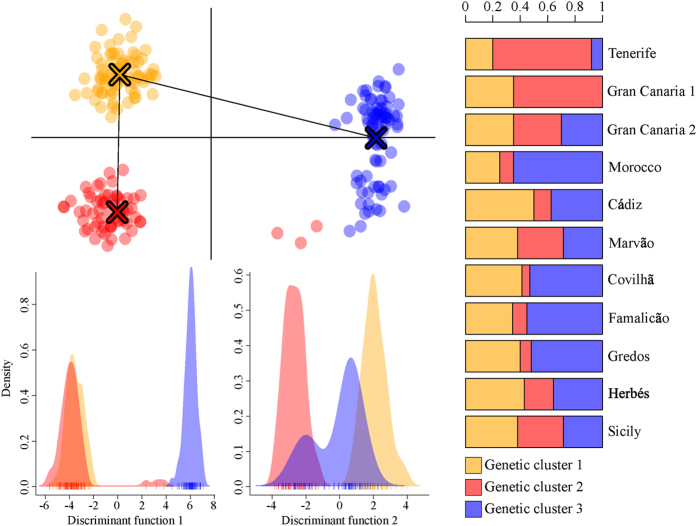
Scatter-plot (**top left**) of the three genetic clusters of *P. carporrhizans* resulting from discriminant analysis of principal components (DAPC). Black lines represent the minimum spanning tree based on the squared distances between clusters within the entire space. **Bottom left:** plot of the densities of individuals on the two retained discriminant functions. **Right:** stacked barplot of cluster distribution for 11 populations of *P. carporrhizans*.

Based on the discriminant analysis of principal components (DAPC), three genetic clusters were considered optimal to describe the data (lowest BIC value, see Fig. S2, Supporting Information). Based on the two retained discriminant functions, the derived probabilities of group membership for each individual were high (>0.95 for each cluster). The three genetic clusters were not geographically restricted (Fig. 1), thus indicating great levels of admixture. Genetic cluster 2 was the most represented gene pool in the island populations, while genetic cluster 3 was the most represented one in the mainland populations. Only in Gran Canaria 1 cluster 3 was missing.

### Spatial analysis

The isolation by distance analysis revealed a clear isolation by distance pattern (r = 0.472, *P* = 0.005). The scatter plot of local densities of distances (Fig. S3, Supporting Information) showed a single consistent cloud of points indicating a continuous cline of genetic differentiation.

We found 29 centroids of microsatellite alleles (13.18% of all alleles) that were significantly geographically restricted (*P* < 0.05; Table S2, Supporting information).

The overall observed mean geographic distance between all shared alleles was 936.2 km and was significantly smaller than the expected mean under the assumption of panmixia (1,055.6 km, *P* < 0.001). The overall observed mean distance between geographically restricted alleles was also significantly smaller than the expected mean (956.5 vs 1,102.7 km), but higher than the overall observed mean geographic distance between all shared alleles thus indicating over-dispersion (Fig. S4).

### Gene flow analysis

We estimated the effective population size and migration rates with MIGRATE-N. The effective population size (θ) of the Macaronesian Islands group (1.16) was considerably smaller than that of the mainland group (16.6). The migration analysis detected high, unidirectional gene flow from the mainland group to Macaronesia (Table S3, Supporting Information).

## Discussion

The Mediterranean Sea became separated from the Atlantic Ocean during the Messinian salinity crisis, approximately 5.5 My ago^28^. While the patterns of genetic isolation between these two basins have been well documented in animals and plants (reviewed in refs 25, 26, 27), very few investigations have been conducted on the genetic diversity of lichen-forming fungi between Macaronesian and Mediterranean populations^17^,^23^. In the present study we analyzed the population genetic structure of the strictly outcrossing lichen-forming fungus *P. carporrhizans* in the Mediterranean region. Sexually reproducing fungal species are expected to show higher genetic diversity and weaker population structure than vegetatively reproducing species^11^,^29^. Our results corroborate this prediction. Using highly variable microsatellite markers we showed that natural populations of *P. carporrhizans* exhibit high levels of genetic diversity and a complete absence of clonality. This is strikingly different from the trend reported for predominantly asexual lichen species, e.g. *L. pulmonaria*, whose populations harbor, almost always, a high number of clonal multilocus genotypes (e.g. refs 17,21).

Allelic richness was comparatively lower in the peripheral populations of the Macaronesian Islands. Macaronesian populations also displayed smaller effective population sizes. These are typical footprints of the so-called founder effect, i.e. the establishment of a new population from a small number of colonizing individuals^30^. Founder events are often followed by a loss of alleles because founders seldom carry all the alleles of the ancestral population. Our results therefore support the ‘abundant-center’ model of population dynamics^31^,^32^ in *P. carporrhizans*, moving from highly diverse and core populations of the Mediterranean Basin towards small and less diverse populations at the edge of the species range.

Our analysis suggests an almost complete lack of population differentiation in *P. carporrhizans*. We found evidence for the existence of three genetic clusters with no clear geographic or ecological structure. In fact all clusters were present in similar frequencies in all investigated regions. As corroborated by the significant isolation by distance, the lack of genetic structure is largely a result of long distance dispersal connecting populations that are continuously distributed across the landscape. The small ascospores of *P. carporrhizans* (10 × 6 μm 33), which could be easily dispersed by wind, may explain the presence of shared alleles even at distances greater than 900 km.

In our study we did not find any evidence for the presence of barriers to dispersal. The absence of dispersal barriers facilitates long distance dispersal thus promoting the disruption of geographic structure in *P. carporrhizans* populations. Furthermore, *P. carporrhizans* inhabits areas such as the Mediterranean Basin and Macaronesian Islands, which are regions that escaped past glacial events^34^. These regions have been suggested to be the glacial refugia for the common phorophytes of *P. carporrhizans*, i.e. *Quercus* spp^35^,^36^,^37^. Repeated glaciation cycles have been shown to cause isolation of populations, followed by speciation^38^. Not experiencing glaciation events would thus have promoted population homogeneity and reduced population subdivision in *P. carporrhizans*.

Another species, for which the genetic variability and population structure have been analyzed using SSR markers, is *Buellia frigida*^19^. In contrast to *P. carporrhizans, Buellia frigida* populations have comparatively higher genetic structure along with high genetic diversity. One of the reasons for the higher population structure in *B. frigida* was suggested to be the limited establishment capability of the spores due to harsh environmental conditions in Antarctica, which hinders inter-population gene flow thus causing population differentiation^19^. Moreover, the reproductive mode of *B. frigida* (self-fertile/homothallic species) is different from *P. carporrhizans*, which might also have contributed to the differences in population structures of the two species. As compared to vegetatively reproducing species however, as expected, *P. carporrhizans* displayed lower genetic structure. For example, a recent study based on sequencing data, showed high population differentiation in the closely-related vegetatively reproducing (isidiate) species, *Parmelina tiliacea*^23^. Interestingly, and unlike *P. carporrhizans*, the Macaronesian populations of *P. tiliacea* were the most genetically diverse.

The high genetic diversity of *P. carporrhizans* indicates high frequency of sexual recombination resulting in novel genotypic combinations in each generation. We obtained evidence for this prediction by analyzing the distribution of mating-type idiomorphs in *P. carporrhizans* populations.

Our results based on mating-type ratios corroborate that *P. carporrhizans* is a heterothallic, i.e. self-incompatible, species, as previously found using RAPD fingerprinting on cultured spore isolates^24^. Moreover our data suggest that recombination is a common event in natural populations of this species as i) we retrieved both mating types in all studied populations, ii) even in populations with significantly skewed mating-type frequencies, values of linkage disequilibrium were close to zero (i.e., fully recombining populations) and clonality was absent. These results confirm the hypothesis put forward by Singh *et al*.^39^ stating that mating-type imbalance cannot be used to predict clonality in populations of lichen-forming fungi. MAT ratios depend in fact upon population history and founder individuals MAT genes. The slightly higher linkage values obtained for some populations may thus be the result of spatial heterogeneity and/or of an origin from a small number of parental thalli. In the case of Cádiz, instead, the significant linkage disequilibrium may simply be an artifact of the limited sample size. In conclusion, the presence of both mating types in each *P. carporrhizans* population seems to be sufficient to ensure panmictic populations and thus low levels of linkage disequilibrium.

Genetic diversity and population structure analyses of *P. carporrhizans* suggested that core populations of the Mediterranean Basin and edge populations in the Macaronesian Islands are still highly connected via gene flow. To corroborate this hypothesis, we estimated migration rates between these two population groups. Our results indicate that populations across the southern range of the species are still highly connected. However, we found that gene flow is unidirectional, i.e., from core towards edge populations. This may well explain the smaller population size and lower allelic richness of the island populations. Moreover, on the Canary Islands, *P. carporrhizans* does not grow at low altitudes, where it is replaced mainly by *Parmotrema* species, but grows in the *Erica*–*Myrica* heath (‘fayal-brezal’) communities on well-lit borders of the laurel forest (‘monteverde’), on sweet chestnut, elms, poplars, pines, etc. from 800 to 1500 m a.s.l. The composition of the forests (at least in Tenerife) has undergone major changes during the Late Holocene, in particular, suitable habitats such as arboreal species of the vegetation alongside laurel forest have considerably declined as a consequence of human activity and climatic variation^40^. On the basis of the limited patches of suitable habitat and the impoverished genetic make-up of the local populations, it can be predicted that island populations are destined to serve only as sink populations.

Based on these observations, we propose that migratory fluxes from core populations can only partially compensate for the detrimental effects of drift, likely caused by founder effects, by maintaining relatively high genetic diversity in peripheral populations. Several studies on different organisms (e.g. refs 31,41, 42, 43) have shown that peripheral populations that are migration-dependent may exhibit lower diversity and smaller population sizes than core populations. Therefore, migration-dependent peripheral populations have been shown to be more vulnerable to the detrimental effects of genetic drift (e.g. ref. 44), a process called extinction-vortex^45^. Our findings of almost unidirectional migration and smaller population size of the edge populations is in accord with the traditional view of islands as the end of the colonization road^46^. The lack of one of the three genetic clusters in a population of the Canary Islands further corroborates this hypothesis.

Future studies including additional peripheral populations, in particular the locally endangered populations of central and northern Europe, would provide additional evidence for the proposed scenario.

## Conclusions

In conclusion, we showed that the strictly sexual, heterothallic lichen-forming fungus *P. carphorrizans* harbors high genetic diversity and connectivity across the Mediterranean region. As a result, we did not find evidence for population structure, suggesting that populations of *P. carphorrizans* act as components of a metapopulation system highly connected by gene flow. The genetic composition of *P. carporrhizans* can thus be explained by a core-edge population model with high migration rates from the Mediterranean Basin (in particular the Iberian Peninsula) towards the Macaronesian Islands.

The apparent absence of dispersal barriers found in our study implies that the establishment of newly dispersed fungal spores is not limited by the availability of compatible photobionts^20^. This, combined with high levels of mycobiont diversity and of recombination within populations, leads to the expectation of high local availability and high levels of horizontal transmission of the photosynthetic partners^21^. As a result, one would expect incongruent fungal-algal population structures^17^,^47^,^48^,^49^,^50^. *Parmelina carporrhizans* represents an ideal system to test these predictions for strictly outcrossing lichen-forming fungi. Additional studies in this direction can further shed light on the effect of dispersal mode on the co-phylogeographic dynamics of the lichen symbiosis.

## Material and Methods

### Sampling collection

**Figure 2:**
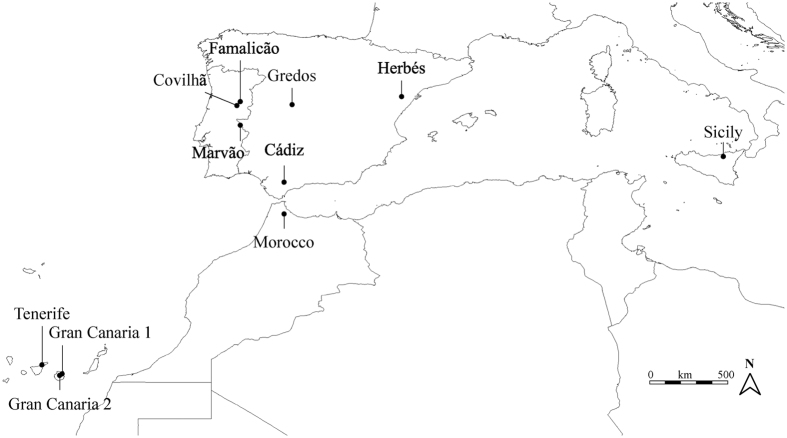
Map of sampling locations of *Parmelina carporrhizans*. The map was generated using DIVA-GIS v7.5 (http://www.diva-gis.org/).

We sampled *P. carporrhizans* from 11 localities to extensively cover the south-western distribution range of the species, from the Macaronesian Islands and Northern Africa, to Southwestern Europe where the species is most abundant (Fig. 2). From each population we collected 8–29 samples (mean 20) for a total of 220 individuals (Table 1). In smaller populations, we collected samples from all colonized trees within the forest stand. In larger populations, we sampled up to ten nearest neighbor trees from the geographic center of a population. From each tree, we collected up to three thalli from different branches or from the trunk. Samples were air dried until further treatment. A list of the samples included in this study is given in Supplementary Table S1, together with voucher information.

### DNA extraction and PCR conditions

Small pieces (0.5 cm^2^) of terminal lobes of freshly collected thalli or recent herbarium specimens were used for DNA isolation with the DNeasy Plant Minikit (Qiagen, Hilden, Germany).

We genotyped each specimen at eight fungus-specific microsatellite markers (Pcar1–8). Details of the markers, PCR conditions and multiplexing are given in ref. 51. Fragment lengths were determined on a 3730 DNA Analyzer (Applied Biosystems, Foster City, CA, USA). Electropherograms were analyzed with Geneious7.1.9^52^ using LIZ-500 as internal size standard. Missing data were ignored in all subsequent analyses except the analysis of population genetic structure (see below, DAPC). For the analysis of population genetic structure, missing data were replaced with the mean frequency of the corresponding allele computed on the whole set of individuals to avoid adding artefactual between-group differentiation^53^.

### MAT genes: primers design and amplification

For designing *P. carporrhizans*-specific MAT-genes primers we obtained partial MAT1-1 and MAT1-2 sequences from the scaffolds of the *P. carporrhizans* genome^51^ via blastx using as query mating-type sequences of *Xanthoria elegans, Xanthoria polycarpa*^54^, and *Lobaria pulmonaria*^39^. Primers were designed with Primer 3 Plus^55^. The newly designed MAT1-1 primers (Alpha-F: GATCAGCCTCGTTCAACCAT, Alpha-R: TAGTGTGCAGGCTCGATGAC) amplified a 389-bp fragment around the alpha-box of the MAT1-1 gene. The MAT1-2 primers (HMG-F: AAGAAGACAAGGTCGCTCGT, HMG-R:CTTGCGAGGCTGGTACTGAT) amplified a 282-bp fragment around the HMG-box of the MAT1-2 gene.

To identify the mating-type idiomorph of each sample, we performed multiplexed PCR reactions in a total volume of 25 μL with 0.65 μL of each MAT1-1 primer (10 μM), 0.6 μL of each MAT1-2 primer (10 μM), 0.4 μL dNTP’s (10 mM; Biotools B&M Labs, Madrid, Spain) and 0.5 μL DNA polymerase (1 U/μL, Biotools B&M Labs, Madrid, Spain) and 2.5 μL of 10–50 ng DNA. PCR reactions were performed with an initial denaturalization at 94 °C for 4 min, followed by 10 cycles of denaturalization step at 95 °C for 10 sec, annealing at 55 to 50 °C for 20 sec and extension at 72 °C for 30 sec, followed by 30 additional cycles with annealing temperature of 50 °C for 20 sec and an additional extension at 72 °C for 5 min. We tested if both idiomorphs were evenly distributed in the entire area and in each individual sampling locality with χ^2^ tests.

### Genetic diversity

Within-population genetic diversity was calculated by estimating allelic richness and private allelic richness using a rarefaction approach, implemented in ADZE^56^. We also calculated Nei’s unbiased haploid diversity (uh) using GenAlEx version 6.41^57^.

To analyze the level of linkage disequilibrium we estimated rBarD (unbiased index of association^58^) within populations and among loci with the function *poppr* of the R package poppr v.2.1.1^59^,^60^. This index detects signatures of multilocus linkage, indicating association between alleles at different loci and clonal reproduction within populations. Significant departure from the null model of no linkage among markers was tested using 999 permutations at a significance level of 0.05.

To quantify genetic differentiation among and within populations we carried out a global analysis of molecular variance (AMOVA) as a weighted average over loci using Arlequin v3.5^61^. Significance was obtained via a non-parametric permutation procedure (20,000 permutations). Additionally, pairwise genetic differences between populations (F_ST_) were calculated using Arlequin v3.5.

### Population genetic structure

To detect population structure we used the multivariate, model-free method DAPC (Discriminant Analysis of Principal Components^53^). We chose to use this method because, contrary to a STRUCTURE-like approach^62^, it is not based on the assumption of unlinked markers and panmictic populations, which are highly unlikely in clonal or partially clonal organism, such as lichens. Clustering on individuals was performed using the R package *adegene*t 2.0.1^53^,^63^,^64^.

### Spatial analysis

We tested for correlation between genetic and geographic distances by performing an isolation by distance (IBD) analysis with the package *adegenet* in R. We used a Mantel test to check for correlation between Edwards’ distances and Euclidean geographic distances among populations with the *mantel.randtest* function. To test whether the correlation between genetic and geographic distances is a result of a continuous or patchy cline of genetic differentiation we plotted local densities of distances to disentangle between the two processes. Local point density was measured using a 2-dimensional kernel density estimation with the function *kde2d* and plotted using a customized color palette with function *image* in the R package MASS^65^. In contrast to a mantel test, which assesses overall correlation of genetic versus geographic distance, the density kernel function looks for underlying genetic structure that may confound or help explain the observed correlation between the two distances.

To further analyze the overall spatial pattern of alleles and test for areas with geographically restricted alleles (GRAs, sensu^17^), we performed spatial analyses of shared alleles (SAShA^66^). This method compares the spatial arrangement of allelic co-occurrences with the expected pattern under panmixia given the same spatial sampling, and it is particularly appropriate for species with high gene flow and subtle genetic differentiation, such as microbes. GRAs were identified following Widmer *et al*.^17^. Briefly, we calculated central geographic x- and y-coordinates (centroids) and the corresponding standard deviations for each allele at each locus. We then used a bootstrap approach randomly subsampling N x and y coordinates (N = no. of individuals carrying a given allele) to infer null distribution and 95% confidence intervals (CI) of each centroid position. Alleles with centroid falling outside the 95% CI were considered as significantly geographically restricted.

For each data set (overall data set, GRAs) we calculated the observed distribution of geographic distances between allelic occurrences in the overall data set and per marker and compared it with the panmictic distribution. To test for significant deviations of the observed mean distances from the expected under panmixia, we performed 1,000 nonparametric permutations of the allele-by-location data sets.

### Analysis of gene flow

To test for the presence and infer the directionality of gene flow between the Macaronesian Islands and the mainland populations, we used the coalescent-based method MIGRATE-N 3.2.6^67^. We estimated mutation scaled immigration rate (M), assuming identical but unknown mutation rates (μ) in all populations. Bayesian estimates of M and θ were obtained under an unconstrained migration model with variable θ for each pair of populations separately. We used a uniform prior on both θ (0.0–0.50) and M (0.0–50). A Metropolis-coupled Monte-Carlo chain with static heating was run for 50 × 10^6^ generations, recording every 100 Kth step after a burn-in period of 5 × 10^4^ generations. Convergence was monitored with Tracer (http://beast.bio.ed.ac.uk). All effective sample sizes of the MCMC chain were larger than 10^4^.

## Additional Information

**How to cite this article**: Alors, D. *et al*. Panmixia and dispersal from the Mediterranean Basin to Macaronesian Islands of a macrolichen species. *Sci. Rep.* **7**, 40879; doi: 10.1038/srep40879 (2017).

**Publisher's note:** Springer Nature remains neutral with regard to jurisdictional claims in published maps and institutional affiliations.

## Supplementary Material

Supplementary Material

## Figures and Tables

**Table 1:** Population genetic parameters for eight microsatellite markers and MAT genes in 11 populations of *P. carporrhizans*.

Population	N	MAT1-1:MAT1-2 (%)	χ^2^	uh mean (st. error)	rBarD	AR mean (st error)	PAR mean (st. error)	Regional AR mean (st. error)	Regional PAR mean (st. error)
Tenerife	25	44:56	0.7	0.744 (0.060)	−0.002	4.256 (0.388)	0.447 (0.122)	11.486 (1.787)	3.095 (0.742)
Gran Canaria 1	20	55:45	1	0.785 (0.042)	**0.045**	4.596 (0.363)	0.531 (0.230)		
Gran Canaria 2	20	59:41	2	0.818 (0.040)	−0.001	4.798 (0.444)	0.489 (0.137)		
Morocco	20	65:35	**4.5**	0.848 (0.054)	−0.004	5.521 (0.572)	0.872 (0.270)	19.067 (2.529)	10.676 (1.465)
Cádiz	8	50:50	0	0.826 (0.030)	**0.288**	3.973 (0.247)	0.551 (0.191)		
Marvão	21	55:45	1	0.859 (0.035)	−0.005	5.524 (0.454)	0.706 (0.171)		
Covilhã	17	59:41	2	0.872 (0.027)	0.000	5.623 (0.310)	0.875 (0.243)		
Famalicão	29	50:50	0	0.874 (0.032)	**0.043**	5.695 (0.361)	0.844 (0.138)		
Gredos	25	47:53	0	0.893 (0.033)	0.005	6.047 (0.433)	1.072 (0.173)		
Herbés	14	73:27	**10.33**	0.886 (0.035)	**0.097**	5.786 (0.524)	0.856 (0.175)		
Sicily	21	55:45	0	0.898 (0.018)	0.008	5.860 (0.280)	0.878 (0.136)		

N: number of samples; MAT1-1:MAT1-2: mating-type idiomorph ratio within population; χ^2^: chi square test of MAT proportions; uh: unbiased haploid genetic diversity; rBarD: unbiased measure of linkage disequilibrium; AR: rarefied allelic richness (N = 8 samples per population, N = 63 per region); PAR = rarefied private allelic richness (N = 8 samples per population, N = 63 per region). Values in bold represent significant *P* values (α = 0.05).

**Table 2:** Global Analysis of Molecular Variance (AMOVA) as a weighted average over 8 loci carried out to estimate *P. carporrhizans* population differentiation.

Source of variation	df	Sum of squares	Variance components	% variation
Among regions	1	23.39	0.20	5.57
Among population within regions	9	44.88	0.08	2.29
Within populations	209	683.13	3.38	92.14
Total	219	751.40	3.67	

**Table 3:** Pairwise F_ST_ values for 11 populations of *P. carporrhizans*.

Population	Tenerife	Gran Canaria 1	Gran Canaria 2	Morocco	Cádiz	Marvão	Covilhã	Famalicão	Gredos	Herbés	Sicily
Tenerife	—	—	—	—	—	—	—	—	—	—	—
Gran Canaria 1	0.002	—	—	—	—	—	—	—	—	—	—
Gran Canaria 2	0.004	0.003	—	—	—	—	—	—	—	—	—
Morocco	0.004	0.003	0.005	—	—	—	—	—	—	—	—
Cádiz	0.018	0.016	0.013	0.019	—	—	.	—	—	—	—
Marvão	0.002	0.000	0.003	0.003	0.016	—	—	—	—	—	—
Covilhã	0.002	0.000	0.003	0.003	0.017	0.000	—	—	—	—	—
Famalicão	0.002	0.000	0.003	0.003	**0.016**	0.000	0.000	—	—	—	—
Gredos	0.002	0.000	0.003	0.003	**0.016**	0.000	0.000	0.000	—	—	—
Herbés	0.007	0.005	0.008	0.008	0.023	0.005	0.005	0.005	0.005	—	—
Sicily	0.002	0.000	0.003	0.003	0.016	0.000	0.000	0.000	0.000	0.005	—

Significant values (20,000 bootstrap iterations) are indicated in bold.
