# Supplementary material for: Panmixia and dispersal from the Mediterranean Basin to Macaronesian Islands of a macrolichen species
Source: Sci Rep. 2017 Jan 19;7:40879. doi: 10.1038/srep40879 (PMC5244402; doi:10.1038/srep40879)

Supplementary Material

Panmixia and dispersal from the Mediterranean basin to Macaronesian islands of a macrolichen species

David Alors*,[1], Francesco Dal Grande*,[2], Paloma Cubas[1], Ana Crespo[1], Imke Schmitt[2,3], M. Carmen Molina[4] and Pradeep K. Divakar[#,1]

[1]Departamento de Biología Vegetal II, Facultad de Farmacia, Universidad Complutense de Madrid, 28040 Madrid, Spain

[2]Senckenberg Biodiversity and Climate Research Centre (BiK-F), Senckenberganlage 25, 60325 Frankfurt am Main, Germany

[3]Institute of Ecology, Evolution and Diversity, Goethe Universität, Max-von-Laue-Str. 13, D-60438 Frankfurt, Germany

[4]Departamento de Biología y Geología, Física y Química Inorgánica (Área de Biodiversidad y Conservación), ESCET, Universidad Rey Juan Carlos, Móstoles, 28933 Madrid, Spain

*These authors contributed equally

Corresponding author: Pradeep K. Divakar, email: pdivakar@farm.ucm.es

Supplementary Table 1: Voucher information and multilocus genotypes (8 microsatellite markers) for 220 *P. carporrhizans* thalli from 11 populations.

Population	DNA	Pcar1	Pcar4	Pcar2	Pcar3	Pcar5	Pcar8	Pcar6	Pcar7	Longitude	Latitude	Altitude	Phorophyte
Tenerife	3678*	137	295	264	188	229	427	213	145	-16.4153	28.4533	894	*Castanea sativa*
Tenerife	3679*	137	315	262	239	249	371	213	205	-16.4153	28.4533	894	*Castanea sativa*
Tenerife	3680*	137	290	264	182	243	432	219	155	-16.4153	28.4533	894	*Castanea sativa*
Tenerife	3681*	137	290	262	188	227	432	219	174	-16.4153	28.4533	894	*Castanea sativa*
Tenerife	3682*	123	300	262	239	272	432	219	202	-16.4153	28.4533	894	*Castanea sativa*
Tenerife	3683*	147	310	264	212	272	432	219	202	-16.4153	28.4533	894	*Castanea sativa*
Tenerife	3684*	135	320	262	191	262	371	216	211	-16.4153	28.4533	894	*Castanea sativa*
Tenerife	3685*	137	295	262	182	-	432	219	145	-16.4153	28.4533	894	*Castanea sativa*
Tenerife	3686*	137	295	264	191	272	432	207	211	-16.4153	28.4533	894	*Castanea sativa*
Tenerife	3687*	137	290	264	236	272	371	213	211	-16.4153	28.4533	894	*Castanea sativa*
Tenerife	3688	139	310	248	197	229	432	213	148	-16.4153	28.4533	894	*Castanea sativa*
Tenerife	3689	137	315	260	191	229	381	219	223	-16.4153	28.4533	894	*Castanea sativa*
Tenerife	3690	137	290	262	239	229	381	219	148	-16.4153	28.4533	894	*Castanea sativa*
Tenerife	4369*	137	290	248	185	249	432	219	197	-16.4154	28.4513	895	*Prunus cerasus*
Tenerife	4370	137	290	248	239	243	432	216	205	-16.4154	28.4513	895	*Prunus cerasus*
Tenerife	4371	137	290	260	191	249	435	219	216	-16.4154	28.4513	895	*Prunus cerasus*
Tenerife	4372	137	315	262	185	229	381	251	205	-16.4154	28.4513	895	*Prunus cerasus*
Tenerife	4373	137	315	262	239	243	371	219	171	-16.4154	28.4513	895	*Prunus cerasus*
Tenerife	4374	137	315	262	185	229	381	251	205	-16.4154	28.4513	895	*Prunus cerasus*
Tenerife	4375	137	295	262	236	243	371	270	148	-16.4154	28.4513	895	*Prunus cerasus*
Tenerife	4376	137	315	260	191	229	432	219	148	-16.4154	28.4513	895	*Prunus cerasus*
Tenerife	4377	137	315	248	230	249	432	216	171	-16.4154	28.4513	895	*Prunus cerasus*
Tenerife	4378*	141	290	248	239	229	371	216	123	-16.4154	28.4513	895	*Prunus cerasus*
Tenerife	4379	137	275	260	185	227	432	270	123	-16.4154	28.4513	895	*Prunus cerasus*
Tenerife	4380	137	295	248	185	229	435	219	148	-16.4154	28.4513	895	*Prunus cerasus*
Gran Canaria1	3645*	137	300	264	185	243	393	219	185	-15.5925	27.9892	1552	*Pinus radiata*
Gran Canaria1	3646*	137	295	250	236	229	371	219	185	-15.5925	27.9892	1499	*Pinus radiata*
Gran Canaria1	3647*	137	280	250	236	227	417	219	168	-15.5925	27.9892	1552	*Pinus radiata*
Gran Canaria1	3648*	137	310	262	233	249	414	261	168	-15.5925	27.9892	1552	*Pinus radiata*
Gran Canaria1	3649*	141	295	262	182	227	-	204	120	-15.5925	27.9892	1552	*Pinus radiata*
Gran Canaria1	3650*	137	305	264	212	251	396	246	165	-15.5925	27.9892	1552	*Pinus radiata*
Gran Canaria1	3651*	137	305	264	221	229	396	219	165	-15.5925	27.9892	1552	*Pinus radiata*
Gran Canaria1	3652*	137	315	250	224	229	417	213	120	-15.5925	27.9892	1552	*Pinus radiata*
Gran Canaria1	3653*	141	295	250	236	229	417	213	120	-15.5925	27.9892	1552	*Pinus radiata*
Gran Canaria1	3654*	139	295	250	236	243	408	207	202	-15.5925	27.9892	1552	*Pinus radiata*
Gran Canaria1	3655*	137	295	262	191	241	371	213	202	-15.5925	27.9892	1552	*Pinus radiata*
Gran Canaria1	3656*	123	290	264	191	229	408	210	223	-15.5925	27.9892	1552	*Pinus radiata*
Gran Canaria1	3657*	137	295	262	212	229	408	213	202	-15.5925	27.9892	1552	*Pinus radiata*
Gran Canaria1	3658*	123	295	250	236	229	371	270	202	-15.5925	27.9892	1552	*Pinus radiata*
Gran Canaria1	3659*	137	295	250	236	229	432	213	202	-15.5925	27.9892	1552	*Pinus radiata*
Gran Canaria1	3660*	139	315	264	236	229	432	246	220	-15.5925	27.9892	1552	*Pinus radiata*
Gran Canaria1	3661*	137	280	264	233	229	432	204	220	-15.5925	27.9892	1552	*Pinus radiata*
Gran Canaria1	3662*	137	295	262	248	249	393	261	220	-15.5925	27.9892	1552	*Pinus radiata*
Gran Canaria1	3663*	137	315	262	182	229	371	261	145	-15.5925	27.9892	1552	*Pinus radiata*
Gran Canaria1	3664*	141	290	206	242	249	432	261	211	-15.5925	27.9892	1552	*Pinus radiata*
Gran Canaria2	3619	135	295	262	212	229	371	213	214	-15.5414	28.0003	945	*Prunus cerasus*
Gran Canaria2	3620	123	265	262	185	243	432	225	177	-15.5414	28.0003	945	*Prunus cerasus*
Gran Canaria2	3621	141	300	262	236	229	393	219	205	-15.5414	28.0003	945	*Prunus cerasus*
Gran Canaria2	3622	137	275	262	242	241	393	219	148	-15.5414	28.0003	945	*Prunus cerasus*
Gran Canaria2	3623	123	275	260	191	249	371	219	123	-15.5414	28.0003	945	*NA*
Gran Canaria2	3624	137	315	248	212	241	420	213	-	-15.5414	28.0003	945	*NA*
Gran Canaria2	3625	123	295	248	236	229	371	216	211	-15.5414	28.0003	945	*Prunus cerasus*
Gran Canaria2	3626	137	315	262	239	227	432	213	148	-15.5414	28.0003	945	*Prunus cerasus*
Gran Canaria2	3627	137	280	262	239	229	393	-	205	-15.5414	28.0003	945	*NA*
Gran Canaria2	3628*	123	295	262	224	229	371	261	171	-15.5414	28.0003	945	*Prunus cerasus*
Gran Canaria2	3629	123	295	260	185	227	371	213	182	-15.5414	28.0003	945	*NA*
Gran Canaria2	3630	123	315	260	194	249	432	225	223	-15.5414	28.0003	945	*NA*
Gran Canaria2	3631	137	295	260	236	229	417	207	205	-15.5414	28.0003	945	*Chamaecytisus proliferus*
Gran Canaria2	3636	123	-	262	-	249	420	246	-	-15.5414	28.0003	945	*NA*
Gran Canaria2	3637	137	320	248	236	227	408	261	123	-15.5414	28.0003	1329	*Ulmus sp.*
Gran Canaria2	3638*	123	320	262	236	249	420	246	205	-15.5414	28.0003	1329	*Ulmus sp.*
Gran Canaria2	3639	123	290	260	242	227	427	213	171	-15.5414	28.0003	1329	*NA*
Gran Canaria2	3641	123	280	260	200	216	432	219	225	-15.5414	28.0003	1329	*NA*
Gran Canaria2	3642	139	275	260	-	-	-	216	171	-15.5414	28.0003	1329	*NA*
Gran Canaria2	3668	137	330	262	230	229	374	213	223	-15.5414	28.0003	1329	*NA*
Morocco	4100	135	305	260	191	245	414	213	-	-5.3742	35.346	701	*Olea europaea*
Morocco	4101	123	305	262	176	-	466	219	-	-5.3742	35.346	701	*Olea europaea*
Morocco	4102	123	300	248	200	245	414	216	168	-5.3742	35.346	701	*Olea europaea*
Morocco	4103	123	300	260	239	229	408	213	157	-5.3742	35.346	701	*NA*
Morocco	4104	123	305	262	176	243	466	219	168	-5.3742	35.346	701	*Olea europaea*
Morocco	4105	123	285	260	188	243	396	210	168	-5.3742	35.346	701	*Olea europaea*

Morocco	4106	145	300	260	176	225	414	210	254	-5.3742	35.346	701	*NA*
Morocco	4107	129	310	260	270	253	417	207	216	-5.3742	35.346	701	*NA*
Morocco	4108	123	285	260	153	229	420	219	197	-5.3873	35.3504	655	*Pinus sp.*
Morocco	4109	123	290	260	182	251	414	249	223	-5.3873	35.3504	655	*Pinus sp.*
Morocco	4110	137	250	260	224	229	406	197	155	-5.3873	35.3504	655	*Pinus sp.*
Morocco	4111	137	300	262	236	225	388	186	123	-5.3873	35.3504	655	*Pinus sp.*
Morocco	4112	141	305	260	233	229	381	179	214	-5.3873	35.3504	655	*Pinus sp.*
Morocco	4114	135	325	260	194	249	411	219	216	-5.3873	35.3504	655	*Pinus sp.*
Morocco	4118	137	275	260	167	251	406	213	234	-5.3873	35.3504	655	*Pinus sp.*
Morocco	4119	123	295	248	191	249	452	219	228	-5.3873	35.3504	655	*Pinus sp.*
Morocco	4120*	123	295	260	194	249	399	219	197	-5.3873	35.3504	655	*Pinus sp.*
Morocco	4122	137	285	262	230	256	411	179	171	-5.3873	35.3504	655	*Pinus sp.*
Morocco	4123	137	290	260	170	249	399	222	182	-5.3873	35.3504	655	*Pinus sp.*
Morocco	4124	123	290	262	251	229	399	207	125	-5.3873	35.3504	655	*Pinus sp.*
Cádiz	4384	123	295	262	191	251	-	-	165	-5.3776	36.7828	1168	*Pinus sp.*
Cádiz	4385	-	285	260	150	229	396	210	182	-5.3776	36.7828	1168	*Pinus sp.*
Cádiz	4387	-	295	262	191	251	359	210	165	-5.3776	36.7828	1168	*Pinus sp.*
Cádiz	4388	141	300	262	182	243	396	179	208	-5.3776	36.7828	1168	*Pinus sp.*
Cádiz	4389*	123	310	240	221	229	399	204	202	-5.3776	36.7828	1168	*Pinus sp.*
Cádiz	4390	137	305	250	188	243	381	197	157	-5.3776	36.7828	1168	*Pinus sp.*
Cádiz	4392*	127	285	260	150	229	381	210	194	-5.3776	36.7828	1168	*Pinus sp.*
Cádiz	4421	127	285	260	153	229	396	210	182	-5.3776	36.7828	1168	*Pinus sp.*
Marvão	4492	123	310	260	197	251	396	216	165	-7.3785	39.3951	858	*Castanea sativa*
Marvão	4493	137	310	262	173	251	393	222	145	-7.3785	39.3951	858	*Castanea sativa*
Marvão	4494	143	300	262	167	251	417	173	-	-7.3785	39.3951	858	*Castanea sativa*
Marvão	4495	141	320	262	173	249	404	213	162	-7.3785	39.3951	858	*Castanea sativa*
Marvão	4496*	137	290	260	182	249	427	216	165	-7.3785	39.3951	858	*Castanea sativa*
Marvão	4497	137	275	284	206	243	396	216	115	-7.3785	39.3951	858	*Castanea sativa*
Marvão	4498	137	280	248	176	251	396	279	188	-7.3785	39.3951	858	*Castanea sativa*
Marvão	4499	133	300	262	212	229	396	210	157	-7.3785	39.3951	858	*Castanea sativa*
Marvão	4500	135	285	260	188	243	411	207	177	-7.3785	39.3951	858	*Castanea sativa*
Marvão	4501	157	260	262	197	251	441	184	208	-7.3785	39.3951	858	*Castanea sativa*
Marvão	4502	137	320	262	170	227	406	210	162	-7.3785	39.3951	858	*Castanea sativa*
Marvão	4503	137	325	248	194	251	408	207	157	-7.3785	39.3951	858	*Castanea sativa*
Marvão	4504	137	240	266	209	243	393	210	125	-7.3788	39.3995	711	*Ulmus sp.*
Marvão	4505	143	290	262	221	251	420	194	-	-7.3788	39.3995	711	*Ulmus sp.*
Marvão	4506	123	310	262	185	251	374	213	145	-7.3788	39.3995	711	*Ulmus sp.*
Marvão	4507	137	305	260	185	253	439	216	123	-7.3788	39.3995	711	*Ulmus sp.*
Marvão	4508*	139	310	260	197	249	-	225	162	-7.3788	39.3995	711	*Ulmus sp.*
Marvão	4509	123	320	262	191	249	-	207	162	-7.3788	39.3995	711	*Ulmus sp.*
Marvão	4510	141	290	266	230	251	396	219	125	-7.3788	39.3995	711	*Ulmus sp.*
Marvão	4511	137	275	260	284	227	411	216	157	-7.3788	39.3995	711	*Ulmus sp.*
Marvão	4512	137	305	262	254	243	-	216	145	-7.3788	39.3995	711	*Ulmus sp.*
Covilhã	4513	123	310	260	176	243	-	204	185	-7.5236	40.2863	990	*Quercus robur*
Covilhã	4514	137	315	260	185	251	439	216	159	-7.5236	40.2863	990	*Quercus robur*
Covilhã	4515	-	330	262	206	249	399	279	157	-7.5236	40.2863	990	*Quercus robur*
Covilhã	4516*	141	300	260	197	243	-	204	157	-7.5236	40.2863	990	*Quercus robur*
Covilhã	4517	143	330	242	185	253	396	179	188	-7.5236	40.2863	990	*Quercus robur*
Covilhã	4518	123	275	280	185	253	414	204	188	-7.5236	40.2863	990	*Quercus robur*
Covilhã	4519	141	295	260	203	251	406	207	191	-7.5236	40.2863	990	*Quercus robur*
Covilhã	4520	141	325	260	191	243	402	204	125	-7.5236	40.2863	990	*Quercus robur*
Covilhã	4521	123	300	260	236	225	406	216	159	-7.5236	40.2863	990	*Quercus robur*
Covilhã	4522*	135	290	260	191	251	411	204	157	-7.5236	40.2863	990	*Quercus robur*
Covilhã	4523	139	315	282	170	249	411	222	174	-7.5236	40.2863	990	*Quercus robur*
Covilhã	4524	123	320	266	197	229	406	204	155	-7.5236	40.2863	990	*Quercus robur*
Covilhã	4525	253	290	260	185	253	417	279	314	-7.5236	40.2863	990	*Quercus robur*
Covilhã	4526	123	310	272	167	227	399	216	151	-7.5236	40.2863	990	*Quercus robur*
Covilhã	4527	123	310	268	185	251	396	207	251	-7.5236	40.2863	990	*Quercus robur*
Covilhã	4528	137	290	262	185	229	-	222	162	-7.5236	40.2863	990	*Quercus robur*
Covilhã	4529	137	250	260	188	253	383	210	112	-7.5236	40.2863	990	*Quercus robur*
Famalicão	4530	123	270	260	188	249	-	216	151	-7.3697	40.4463	817	*Quercus sp.*
Famalicão	4531	137	250	260	197	229	-	207	145	-7.3697	40.4463	817	*Quercus sp.*
Famalicão	4532*	127	300	248	182	247	396	197	237	-7.3697	40.4463	817	*Quercus sp.*
Famalicão	4533	135	300	262	206	229	408	213	145	-7.3697	40.4463	817	*Quercus sp.*
Famalicão	4534	123	300	258	200	256	-	186	110	-7.3697	40.4463	817	*Quercus sp.*
Famalicão	4535	137	320	282	218	249	-	204	157	-7.3697	40.4463	817	*Quercus sp.*
Famalicão	4536	141	305	260	200	227	-	210	157	-7.3697	40.4463	817	*Quercus sp.*
Famalicão	4537	137	310	244	185	229	396	207	162	-7.3697	40.4463	817	*Quercus sp.*
Famalicão	4538	141	305	260	153	247	404	207	123	-7.3697	40.4463	817	*Quercus sp.*
Famalicão	4539*	137	245	248	197	256	393	210	113	-7.3697	40.4463	817	*Quercus sp.*
Famalicão	4540	127	330	260	188	259	-	207	157	-7.3697	40.4463	817	*Quercus sp.*
Famalicão	4541	123	265	262	182	-	385	-	-	-7.3697	40.4463	817	*Quercus sp.*
Famalicão	4542	139	250	262	185	227	359	-	-	-7.3697	40.4463	817	*Quercus sp.*
Famalicão	4543	153	285	248	203	251	-	210	145	-7.3697	40.4463	817	*Quercus sp.*
Famalicão	4544	123	310	260	194	253	359	207	165	-7.3697	40.4463	817	*Quercus sp.*
Famalicão	4545	133	300	266	179	249	408	191	159	-7.3697	40.4463	817	*Quercus sp.*
Famalicão	4546	137	320	260	215	227	365	207	145	-7.3697	40.4463	817	*Quercus sp.*

Famalicão	4547	123	290	260	191	253	371	207	162	-7.3697	40.4463	817	*Quercus sp.*
Famalicão	4548	123	290	262	185	227	414	213	162	-7.3697	40.4463	817	*Quercus sp.*
Famalicão	4549	123	265	260	203	249	408	191	194	-7.3697	40.4463	817	*Quercus sp.*
Famalicão	4550	164	305	260	209	243	460	207	191	-7.3697	40.4463	817	*Quercus sp.*
Famalicão	4551	123	270	260	215	251	406	216	157	-7.3697	40.4463	817	*Quercus sp.*
Famalicão	4552	141	325	260	188	249	-	197	145	-7.3697	40.4463	817	*Quercus sp.*
Famalicão	4554	145	305	260	218	249	327	232	157	-7.3697	40.4463	817	*Quercus sp.*
Famalicão	4555	137	290	260	206	229	292	207	145	-7.3697	40.4463	817	*Quercus sp.*
Famalicão	4556	-	315	266	194	259	359	216	113	-7.3697	40.4463	817	*Quercus sp.*
Famalicão	4557	137	305	260	197	227	406	207	157	-7.3697	40.4463	817	*Quercus sp.*
Famalicão	4558	143	310	266	194	243	411	213	157	-7.3697	40.4463	817	*Quercus sp.*
Famalicão	4559	135	305	260	167	243	323	191	137	-7.3697	40.4463	817	*Quercus sp.*
Gredos	3350	123	240	260	248	256	437	204	113	-5.0124	40.3234	1363	*Ulmus sp.*
Gredos	3364	141	295	294	164	251	388	207	223	-5.0124	40.3234	1363	*Ulmus sp.*
Gredos	3365	137	270	260	191	243	460	207	162	-5.0124	40.3234	1363	*Ulmus sp.*
Gredos	3366	133	250	262	212	253	399	270	123	-5.0124	40.3234	1363	*Ulmus sp.*
Gredos	3367	123	280	260	203	249	402	184	214	-5.0124	40.3234	1363	*Ulmus sp.*
Gredos	3418	123	325	258	194	229	443	207	125	-5.011	40.3079	1363	*Castanea sativa*
Gredos	3419	137	235	248	179	251	417	204	137	-5.011	40.3079	1363	*Castanea sativa*
Gredos	3420*	141	260	274	227	249	402	207	157	-5.011	40.3079	1363	*Castanea sativa*
Gredos	3421	137	290	260	212	251	406	210	216	-5.011	40.3079	1363	*Castanea sativa*
Gredos	3422*	141	290	260	212	251	408	204	162	-5.011	40.3079	1363	*Castanea sativa*
Gredos	3423	123	305	242	233	251	390	289	157	-5.011	40.3079	1363	*Castanea sativa*
Gredos	3424	141	295	260	182	243	414	204	157	-5.011	40.3079	1363	*Castanea sativa*
Gredos	3425	141	290	260	212	259	396	216	197	-5.011	40.3079	1363	*Castanea sativa*
Gredos	3426	137	300	260	191	251	427	204	145	-5.011	40.3079	1363	*Castanea sativa*
Gredos	3427	123	270	260	182	239	441	219	145	-5.011	40.3079	1363	*Castanea sativa*
Gredos	3428	123	250	262	200	251	404	213	185	-5.011	40.3079	1363	*Castanea sativa*
Gredos	3429	139	280	252	215	249	393	216	168	-5.011	40.3079	1363	*Castanea sativa*
Gredos	3430	123	305	260	239	233	396	194	148	-5.011	40.3079	1363	*Castanea sativa*
Gredos	3431	143	250	262	188	251	411	207	157	-5.011	40.3079	1363	*Castanea sativa*
Gredos	3432	141	315	230	179	229	385	216	245	-5.011	40.3079	1363	*Castanea sativa*
Gredos	3433	137	300	248	242	229	399	228	148	-5.011	40.3079	1363	*Castanea sativa*
Gredos	3434	127	295	260	161	247	408	197	142	-5.011	40.3079	1363	*Castanea sativa*
Gredos	3435	127	255	260	215	229	396	179	177	-5.011	40.3079	1363	*Castanea sativa*
Gredos	3436	143	275	248	182	249	381	173	208	-5.011	40.3079	1363	*Castanea sativa*
Gredos	3437	123	313	260	191	251	404	179	223	-5.011	40.3079	1363	*Castanea sativa*
Herbés	4398	137	265	260	218	251	388	213	-	-0.0227	40.6899	919	*Quercus faginea*
Herbés	4399	137	265	260	218	251	388	213	205	-0.0227	40.6899	919	*Quercus faginea*
Herbés	4400*	141	285	248	221	239	404	170	225	-0.0227	40.6899	919	*Quercus faginea*
Herbés	4401	143	280	274	209	251	423	207	157	-0.0227	40.6899	919	*Quercus faginea*
Herbés	4405	135	295	260	218	247	408	216	237	-0.0227	40.6899	919	*Quercus faginea*
Herbés	4406*	135	285	242	224	229	396	204	223	-0.0227	40.6899	919	*Quercus faginea*
Herbés	4411	137	290	248	179	229	411	219	151	-0.0227	40.6899	919	*Quercus faginea*
Herbés	4416	141	320	262	188	241	414	182	159	-0.0227	40.6899	919	*Quercus faginea*
Herbés	4417	137	300	260	179	229	396	210	182	-0.0227	40.6899	919	*Quercus faginea*
Herbés	4690	135	305	-	-	251	396	179	-	-0.0227	40.6899	919	*Quercus faginea*
Herbés	4691*	137	305	248	194	249	411	270	216	-0.0227	40.6899	919	*Quercus faginea*
Herbés	4694	-	305	262	209	243	399	182	174	-0.0227	40.6899	919	*Quercus faginea*
Herbés	4695	-	335	260	194	251	408	207	145	-0.0227	40.6899	919	*Quercus faginea*
Herbés	4696	137	330	262	206	229	393	219	148	-0.0227	40.6899	919	*Quercus faginea*
Sicily	3983	123	-	248	203	241	404	207	194	14.6271	37.9451	1183	*Quercus sp.*
Sicily	3984	127	290	266	221	241	414	160	165	14.6271	37.9451	1183	*Quercus sp.*
Sicily	3985	125	-	260	200	251	-	207	165	14.6271	37.9451	1183	*Quercus sp.*
Sicily	4011	137	260	260	197	239	399	261	265	14.4889	37.8817	1223	*Quercus sp.*
Sicily	4012	137	295	262	203	251	-	194	205	14.4889	37.8817	1223	*Quercus sp.*
Sicily	4013	133	285	248	197	249	-	207	139	14.4889	37.8817	1223	*Quercus sp.*
Sicily	4014	137	315	262	197	241	-	207	159	14.4889	37.8817	1223	*Quercus sp.*
Sicily	4015	141	320	248	194	229	381	191	162	14.4889	37.8817	1223	*Quercus sp.*
Sicily	4016	137	320	268	194	249	443	204	191	14.4889	37.8817	1223	*Quercus sp.*
Sicily	4053	141	305	260	185	251	396	179	274	13.3863	37.8685	872	*Quercus sp.*
Sicily	4054	123	260	260	197	243	-	210	205	13.3863	37.8685	872	*Quercus sp.*
Sicily	4055	141	320	260	194	251	399	210	157	13.3863	37.8685	872	*Quercus sp.*
Sicily	4056	143	295	250	206	243	378	207	162	13.3863	37.8685	872	*Quercus sp.*
Sicily	4057	141	290	248	215	239	393	160	157	13.3863	37.8685	872	*Quercus sp.*
Sicily	4058	135	250	262	209	229	402	216	123	13.3863	37.8685	872	*Quercus sp.*
Sicily	4059	137	280	248	200	249	414	207	159	13.3863	37.8685	872	*Quercus sp.*
Sicily	4060	137	275	262	185	249	414	201	194	13.3863	37.8685	872	*Quercus sp.*
Sicily	4061	137	325	294	218	229	383	216	188	13.3863	37.8685	872	*Quercus sp.*
Sicily	4062	135	250	242	206	245	396	216	165	13.3863	37.8685	872	*Quercus sp.*
Sicily	4063	137	250	262	155	251	396	204	115	13.3863	37.8685	872	*Quercus sp.*
Sicily	4064	129	310	260	194	233	393	207	113	13.3863	37.8685	872	*Quercus sp.*
Number of alleles/locus	-	16	22	20	36	17	35	28	46	-	-	-	-
% of missing data	-	2.7	1.4	0.4	1.4	1.8	10	1.8	4.6	-	-	-	-

*Details of the specimens kept in MAF Herbarium (Madrid, Spain)

Gran Canaria 2: DNA 3638(MAF-Lich 20664) and 3628 (MAF-Lich 20665), Spain, Gran Canaria, San Mateo, *A. Crespo, P. Cubas, A. Santos and P.K. Divakar*, 23/06/2009. **Tenerife:** DNA 4378(MAF-Lich 20666) and 4369(MAF-Lich 20667), Spain, Tenerife, *A. Sauzal, A. Crespo, P. Crespo and V. J. Rico*, 14/03/2014. **Morocco:** DNA 4120(MAF-Lich 20668) Morocco, Tetuán, Beni Hassan, *D. Alors and C.G. Boluda*, 21/10/2013. **Cádiz**: DNA 4389(MAF-Lich 20669) and 4392(MAF-Lich 20670), Spain, Cádiz, Grazalema, *A. Crespo, J. Fernández de Bobadilla and J. Núñez-Zapata*, 15/10/2010. **Marvão:** DNA 4508(MAF-Lich 20671) and 4496(MAF-Lich 20672), Portugal, Alto Alentejo, Marvão, *D. Alors, A. Crespo, P.K. Divakar, C. Ruibal and V. J. Rico*, 11/06/2014. **Covilhã**: DNA 4516(MAF-Lich 20673) and 4522 (MAF-Lich 20674), Portugal, Beira Baixa, Covilhã, *D. Alors, A. Crespo, P.K. Divakar, C. Ruibal and V. J. Rico*, 12/06/2014. **Famalicão**: DNA 4539(MAF-Lich 20675) and 4532(MAF-Lich 20676), Portugal, Beira Baixa, Famalicão, *D. Alors, A. Crespo, P.K. Divakar, C. Ruibal and V. J. Rico* in 13/06/2014. **Herbés:** DNA 4400(MAF-Lic 20677) and 4406(MAF-Lich 20679) Spain, Castellón, Herbés, *D. Alors and V. Claramonte*, 19/05/2014. DNA 4691 (MAF- Lich 20678), *D. Alors, C. Alors, J. Villagra and N. Perales*, 29/12/2014.

DNA codes 3645 to 3664 (MAF-Lich 19125 to 19142), 3678 to 3687 (MAF-Lich 19143 to 19152) and 3420, 3422 (MAF-Lich 19191 and MAF-Lich 19192) were included in Alors et al. (2014)

Supplementary Table 2: Geographically restricted alleles of *Parmelina carporrhizans* and geographic coordinates of their centroids.

Locus	Allele	Long	Lat
Pcar1	137	-8.63236	33.98925
Pcar4	250	-0.25257	39.10741
Pcar4	295	-10.54506	31.86994
Pcar4	315	-12.29158	31.46331
Pcar2	250	-11.23765	30.06395
Pcar2	264	-15.96650	28.20011
Pcar3	197	-1.45035	38.46678
Pcar3	203	0.30676	39.55485
Pcar3	206	-0.41309	39.57157
Pcar3	218	-0.23685	40.13847
Pcar3	236	-14.53727	29.27917
Pcar3	239	-13.99602	30.23680
Pcar5	229	-9.39365	33.39248
Pcar5	251	-3.18724	39.09053
Pcar5	272	-16.41531	28.45325
Pcar8	371	-15.37119	28.94487
Pcar8	396	-3.92974	38.19653
Pcar8	432	-16.07599	28.26934
Pcar6	207	-2.82068	38.42865
Pcar6	213	-11.34349	32.67785
Pcar6	219	-12.20842	31.28559
Pcar6	246	-15.56697	27.99472
Pcar7	120	-15.59250	27.98917
Pcar7	148	-12.32043	31.95665
Pcar7	159	0.90593	39.57651
Pcar7	171	-14.14040	29.37563
Pcar7	202	-14.52135	29.20439
Pcar7	211	-16.07597	28.26984
Pcar7	220	-15.59250	27.98917

Supplementary Table3: MIGRATE Bayesian Analysis posterior distribution table.

Parameter	2.5%	25.0%	75.0%	97.5%	Mean
Θ1	0.17	0.73	1.53	2.1	1.16
Θ2	9.13	11.7	15.9	21.33	16.59
M2->1	3.2	3.97	5.07	5.7	8.39
M1->2	0.03	0.53	1.3	1.83	0.94

1: Macaronesian Islands populations; 2: Mainland populations (Morocco, Iberian Peninsula, Sicily); Θ: mutation-scaled effective population size; M: mutation-scaled immigration rate.

Supplementary Figure 1: An example of gel electrophoresis of the PCR products of the mating-type PCR analysis on 29 thalli of *P. carporrhizans* (Famalicão 4534-4559 and Gran Canaria2 3620, 3625-3626). Molecular ruler: AmpliSize 50–2,000 bp (BioRad). Stain: SYBR Safe (Invitrogen, USA).

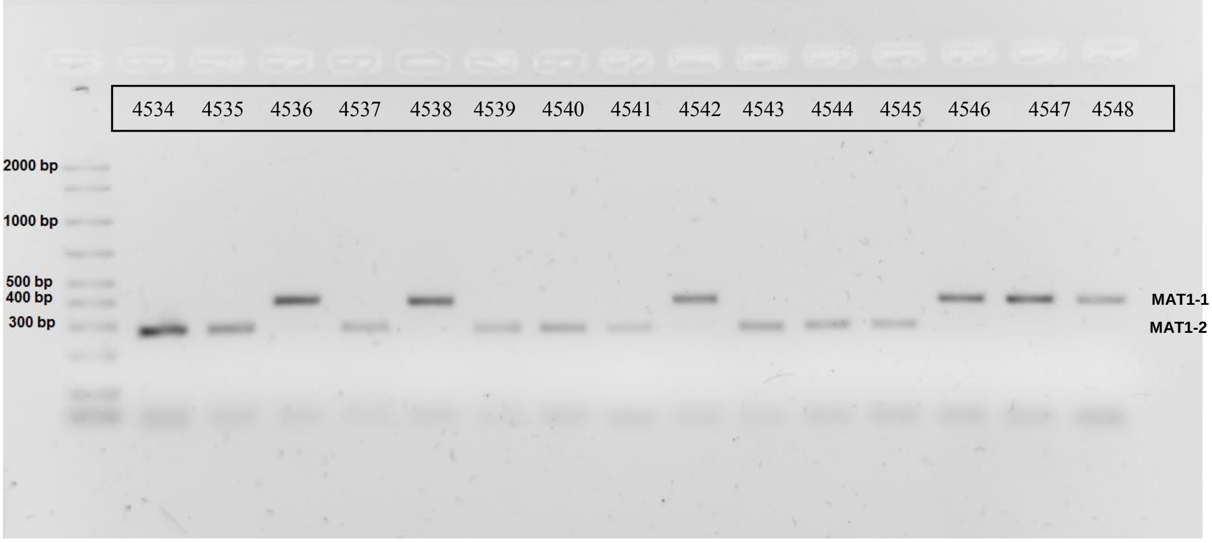

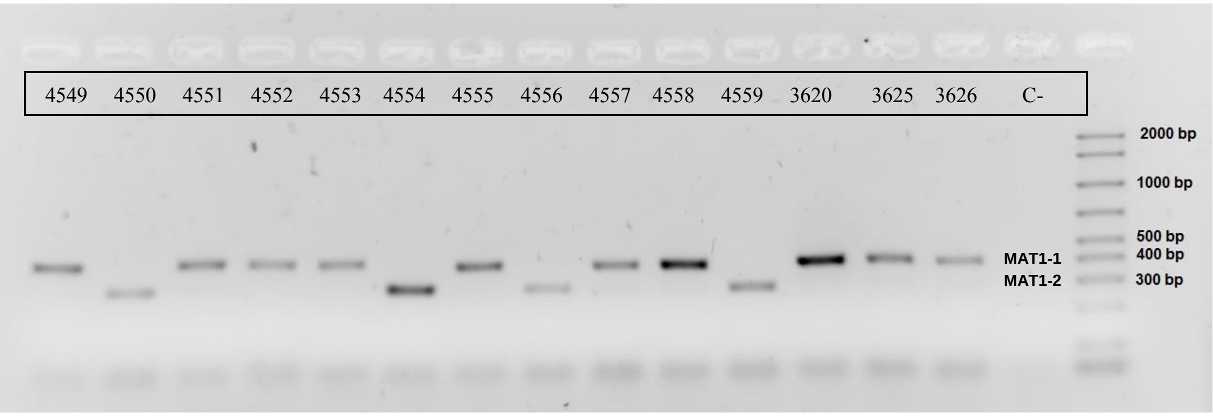

Supplementary Figure 2: Inference of the number of clusters in the DAPC performed on 220 samples of *P. carporrhizans*. K value = 3 (the lowest BIC value) represents the best summary of the data.

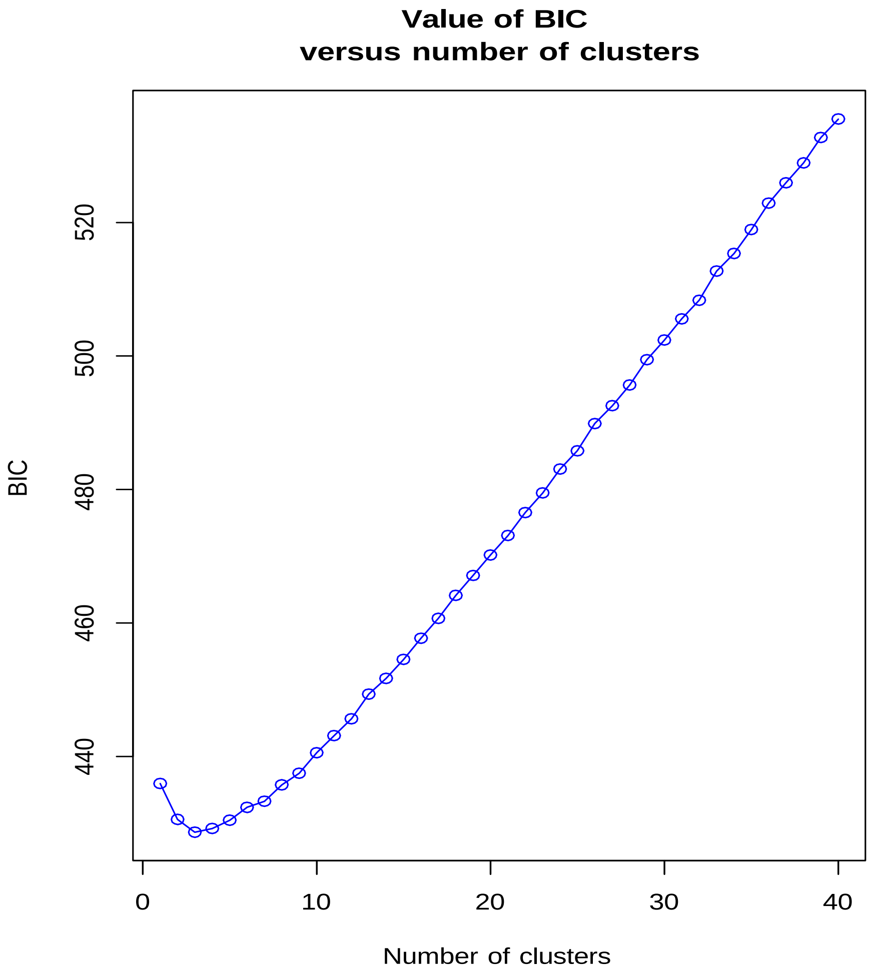

Supplementary Figure 3: Isolation by distance plot illustrating a continuous cline of genetic differentiation in *P. carporrhizans* populations, using a 2-dimensional kernel density estimation in MASS program.

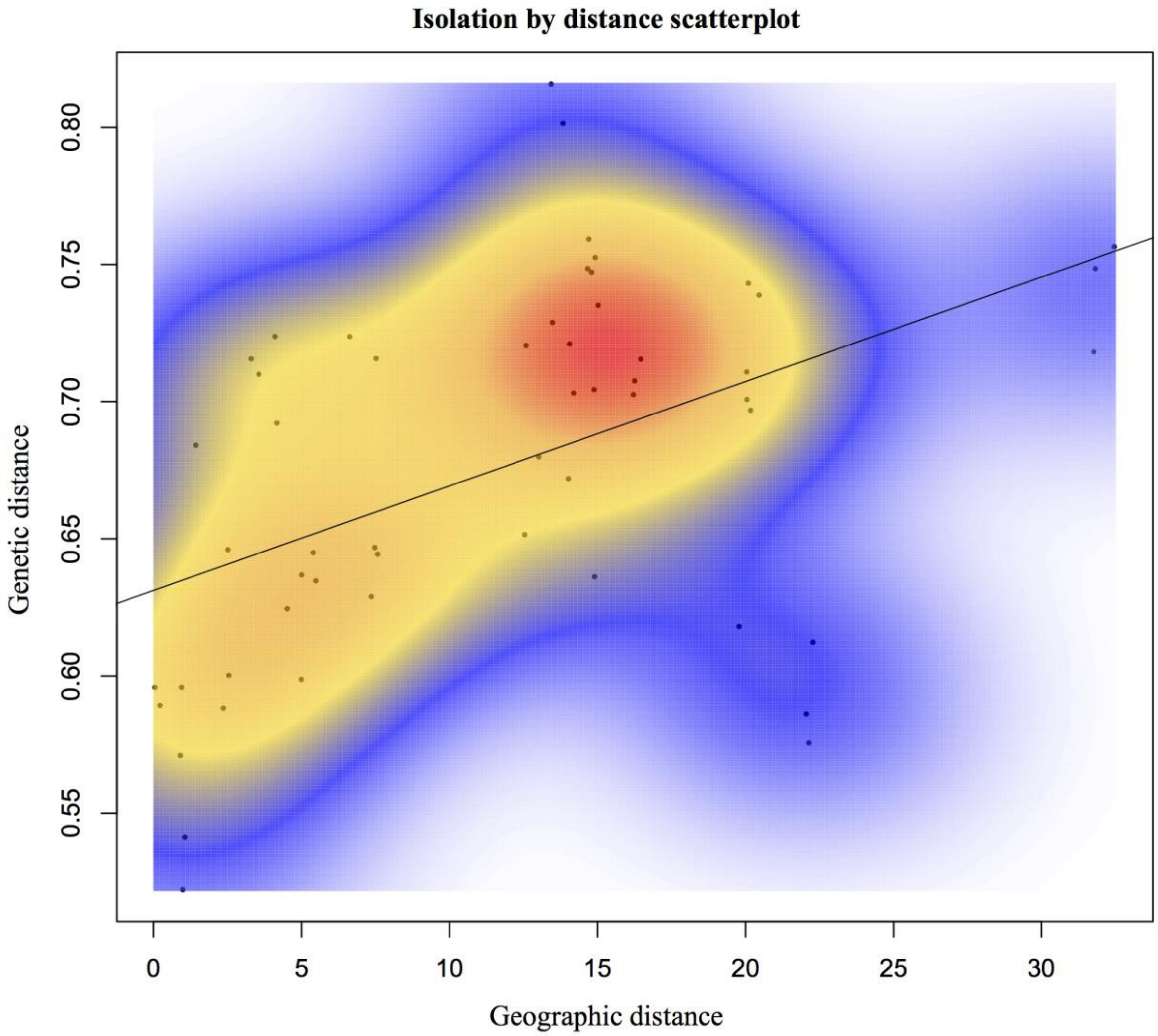

Supplementary Figure 4: Spatial distribution analysis of a) all, and b) geographically restricted shared alleles of *P. carporrhizans*, performed with SAShA. Tests for significant deviations of the observed mean distances from the expected under panmixia were performed with 1,000 nonparametric permutations of the allele-by-location data sets.

a)

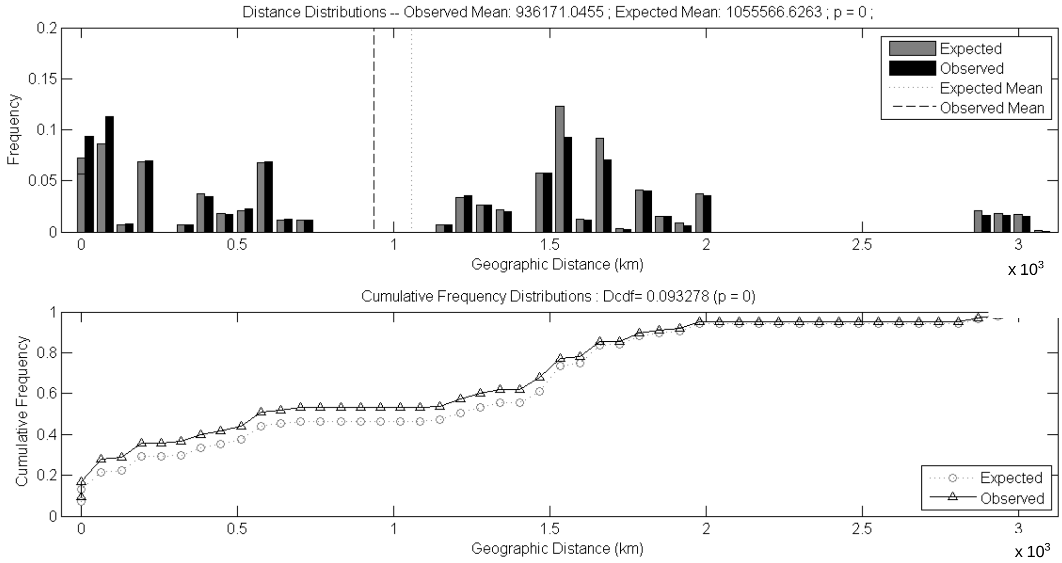

b)

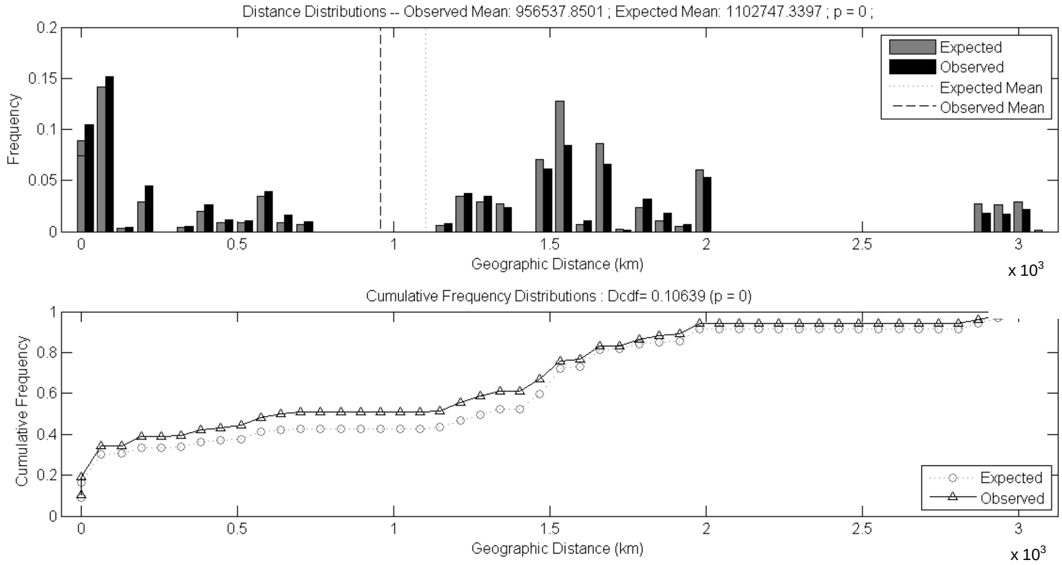

Supplement: Supplementary Material [file srep40879-s1.pdf]
